# Supplementary material for: Discovery and Use of Long dsRNA Mediated RNA Interference to Stimulate Antiviral Protection in Interferon Competent Mammalian Cells
Source: Front Immunol. 2022 May 6;13:859749. doi: 10.3389/fimmu.2022.859749 (PMC9120774; doi:10.3389/fimmu.2022.859749)
Supplement: Supplementary file 1 [file DataSheet_1.pdf]

Supplementary Material

A. GFP

ATGGTGAGCAAGGGCGAGGAGCTGTTCACCGGGGTGGTGCCCATCCTGGTCGAGCTGGACGGCGACGTAA
GFP Forward

ACGGCCACAAGTTCAGCGTGTCCGGCGAGGGCGAGGGCGATGCCACCTACGGCAAGCTGACCCTGAAGTT

CATCTGCACCACCGGCAAGCTGCCCGTGCCCTGGCCCACCCTCGTGACCACCCTGACCTACGGCGTGCAG
200 bp Reverse

TGCTTCAGCCGCTACCCCGACCACATGAAGCAGCACGACTTCTTCAAGTCCGCCATGCCCGAAGGCTACG

TCCAGGAGCGCACCATCTTCTTCAAGGACGACGGCAACTACAAGACCCGCGCCGAGGTGAAGTTCGAGGG
300 bp Reverse

CGACACCCTGGTGAACCGCATCGAGCTGAAGGGCATCGACTTCAAGGAGGACGGCAACATCCTGGGGCAC
400 bp Reverse

AAGCTGGAGTACAACTACAACAGCCACAACGTCTATATCATGGCCGACAAGCAGAAGAACGGCATCAAGG

TGAACTTCAAGATCCGCCACAACATCGAGGACGGCAGCGTGCAGCTCGCCGACCACTACCAGCAGAACAC
500 bp Reverse

CCCCATCGGCGACGGCCCCGTGCTGCTGCCCGACAACCACTACCTGAGCACCCAGTCCGCCCTGAGCAAA
600 bp Reverse

GACCCCAACGAGAAGCGCGATCACATGGTCCTGCTGGAGTTCGTGACCGCCGCCGGGATCACTCTCGGCA

TGGACGAGCTGTACAAGTAA
700 bp Reverse

B. mCherry

ATGGTGAGCAAGGGCGAGGAGGATAACATGGCCATCATCAAGGAGTTCATGCGCTTCAAGGTGCACATGG
mCherry Forward

AGGGCTCCGTGAACGGCCACGAGTTCGAGATCGAGGGCGAGGGCGAGGGCCGCCCCTACGAGGGCACCCA

GACCGCCAAGCTGAAGGTGACCAAGGGTGGCCCCCTGCCCTTCGCCTGGGACATCCTGTCCCCTCAGTTC

ATGTACGGCTCCAAGGCCTACGTGAAGCACCCCGCCGACATCCCCGACTACTTGAAGCTGTCCTTCCCCG

AGGGCTTCAAGTGGGAGCGCGTGATGAACTTCGAGGACGGCGGCGTGGTGACCGTGACCCAGGACTCCTC

CCTGCAGGACGGCGAGTTCATCTACAAGGTGAAGCTGCGCGGCACCAACTTCCCCTCCGACGGCCCCGTA

ATGCAGAAGAAGACCATGGGCTGGGAGGCCTCCTCCGAGCGGATGTACCCCGAGGACGGCGCCCTGAAGG

GCGAGATCAAGCAGAGGCTGAAGCTGAAGGACGGCGGCCACTACGACGCTGAGGTCAAGACCACCTACAA

GGCCAAGAAGCCCGTGCAGCTGCCCGGCGCCTACAACGTCAACATCAAGTTGGACATCACCTCCCACAAC

GAGGACTACACCATCGTGGAACAGTACGAACGCGCCGAGGGCCGCCACTCCACCGGCGGCATGGACGAGC
700 bp Reverse

TGTACAAGTAG

C. β-lac

ATGAGTATTCAACATTTCCGTGTCGCCCTTATTCCCTTTTTTGCGGCATTTTGCCTTCCTGTTTTTGCTC

ACCCAGAAACGCTGGTGAAAGTAAAAGATGCTGAAGATCAGTTGGGTGCACGAGTGGGTTACATCGAACT
β-lac Forward

GGATCTCAACAGCGGTAAGATCCTTGAGAGTTTTCGCCCCGAAGAACGTTTTCCAATGATGAGCACTTTT

AAAGTTCTGCTATGTGGCGCGGTATTATCCCGTATTGACGCCGGGCAAGAGCAACTCGGTCGCCGCATAC

ACTATTCTCAGAATGACTTGGTTGAGTACTCACCAGTCACAGAAAAGCATCTTACGGATGGCATGACAGT

AAGAGAATTATGCAGTGCTGCCATAACCATGAGTGATAACACTGCGGCCAACTTACTTCTGACAACGATC

GGAGGACCGAAGGAGCTAACCGCTTTTTTGCACAACATGGGGGATCATGTAACTCGCCTTGATCGTTGGG

AACCGGAGCTGAATGAAGCCATACCAAACGACGAGCGTGACACCACGATGCCTGTAGCAATGGCAACAAC

GTTGCGCAAACTATTAACTGGCGAACTACTTACTCTAGCTTCCCGGCAACAATTAATAGACTGGATGGAG

GCGGATAAAGTTGCAGGACCACTTCTGCGCTCGGCCCTTCCGGCTGGCTGGTTTATTGCTGATAAATCTG

GAGCCGGTGAGCGTGGGTCTCGCGGTATCATTGCAGCACTGGGGCCAGATGGTAAGCCCTCCCGTATCGT

AGTTATCTACACGACGGGGAGTCAGGCAACTATGGATGAACGAAATAGACAGATCGCTGAGATAGGTGCC

TCACTGATTAAGCATTGGTAAC
750 bp Reverse

Supplementary Figure S1. Binding locations of primers to plasmid sequences for GFP, mCherry and β-lac

Binding locations for the T7 primers that were used to create dsRNA from the positive (GFP) and negative (mCherry and β-lac) control sequences that were found on plasmids.

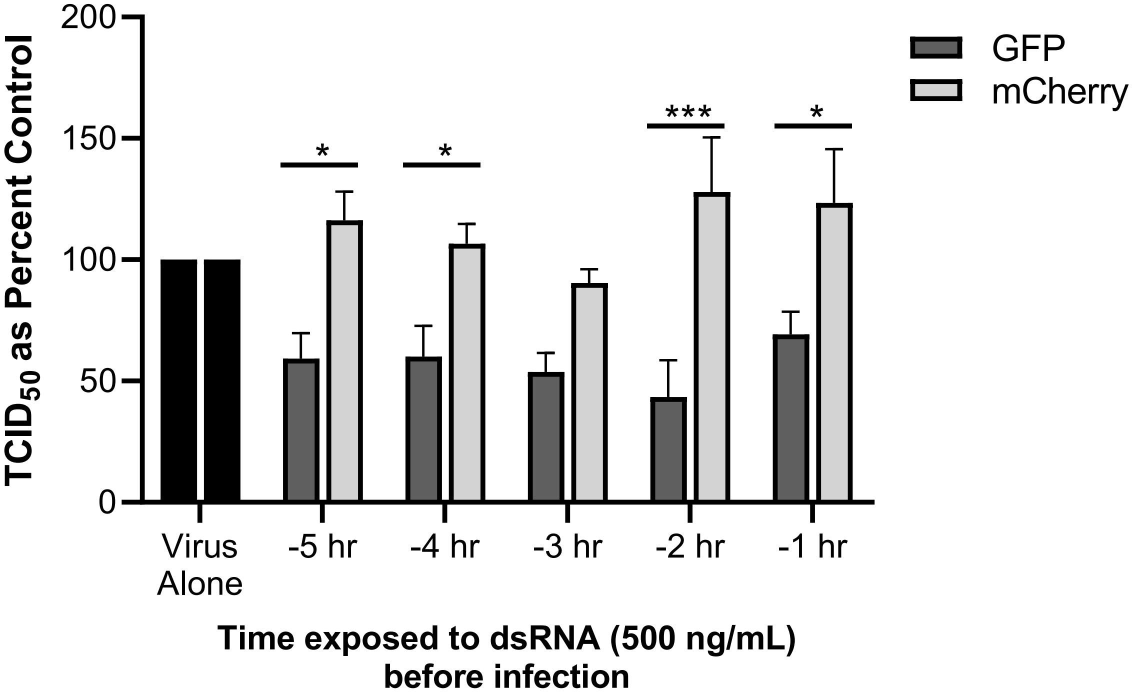

Supplementary Figure S2. dsRNA effective at limiting virus VSV-GFP replication 1-5h prior to infection and at the time of infection.

M14 Cells (75,000 cells/well) were exposed to 500 ng/mL of each dsRNA (700 bp each) at various times before infection with VSV-GFP (MOI = 1). Following 24 hours of infection, supernatants were collected and the TCID50 was calculated using HEL-299 cells. This has been repeated three times. Significant differences were assessed between mCherry and GFP at each individual timepoint using a Sidak's multiple comparisons test.

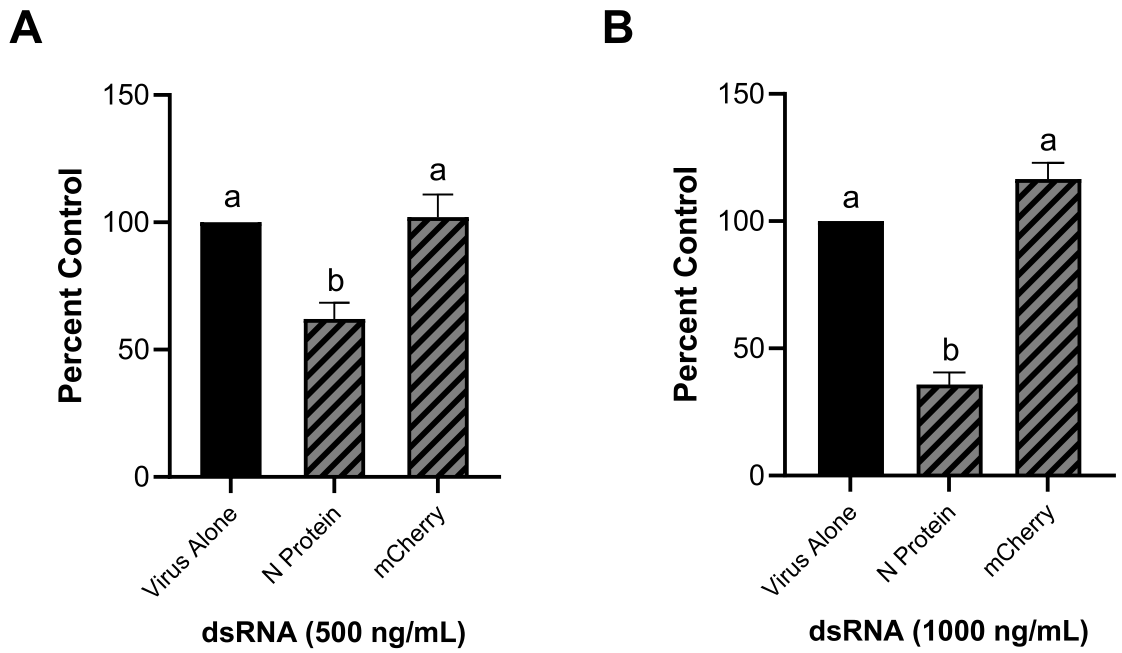

Supplemental Figure S3. Knockdown of VSV-GFP with sequence specific (N Protein) or mis-matched (mCherry) dsRNA using the adsorption method of infection.

THF cells (50,000 cells/well of 24 well plate) were exposed to dsRNA for two hours before infection with VSV-GFP (MOI = 0.1). The virus was allowed to adsorb to the cells for 2h before the media was removed, cells were washed twice with PBS, and 500 µL of fresh, 2% FBS DMEM media was added to each well. After 24h the supernatants were collected, and dilutions completed so that the $TCID_{50}$ could be determined. This method removed any dsRNA from the cells ensuring no dsRNA was present in the supernatants used for quantifying virus titers.
